# Aerosol Forcing Masks and Delays the Formation of the North Atlantic Warming Hole by Three Decades

**DOI:** 10.1029/2020GL090778

**Published:** 2020-11-18

**Authors:** Guy Dagan, Philip Stier, Duncan Watson‐Parris

**Affiliations:** ^1^ Atmospheric, Oceanic and Planetary Physics, Department of Physics University of Oxford Oxford UK

**Keywords:** aerosol, North Atlantic, warming hole, GHGs, AMOC

## Abstract

The North Atlantic warming hole (NAWH) is referred to as a reduced warming, or even cooling, of the North Atlantic during an anthropogenic‐driven global warming. A NAWH is predicted by climate models during the 21st century, and its pattern is already emerging in observations. Despite the known key role of the North Atlantic surface temperatures in setting the Northern Hemisphere climate, the mechanisms behind the NAWH are still not fully understood. Using state‐of‐the‐art climate models, we show that anthropogenic aerosol forcing opposes the formation of the NAWH (by leading to a local warming) and delays its emergence by about 30 years. In agreement with previous studies, we also demonstrate that the relative warming of the North Atlantic under aerosol forcing is due to changes in ocean heat fluxes, rather than air‐sea fluxes. These results suggest that the predicted reduction in aerosol forcing during the 21st century may accelerate the formation of the NAWH.

## Key Points

- Using CMIP6 and CESM‐LE simulations, we show that aerosol forcing generates a global cooling trend with a warming in the North Atlantic
- This trend opposes the North Atlantic warming hole trend due to greenhouse gases
- Aerosol forcing delays the formation of the North Atlantic warming hole by about 30 years

## 1 Introduction

The North Atlantic surface temperature plays a key role in the Northern Hemisphere's climate (O'Reilly et al., 2017; Woollings et al., 2012; Zhang & Delworth, 2006). While climate models predict a global increase in temperature due to anthropogenic emissions of greenhouse gases (GHGs), the surface temperature in the North Atlantic is predicted to decrease due to changes in the ocean meridional heat flux (Caesar et al., 2018; Chemke et al., 2020; Cheng et al., 2013; Drijfhout et al., 2012; Gervais et al., 2018; Manabe & Stouffer, 1993; Marshall et al., 2015; Menary & Wood, 2018; Piecuch et al., 2017; Rahmstorf et al., 2015; Robson et al., 2016; Woollings et al., 2012). The meridional heat flux change could be driven by a decline of the low‐latitude Atlantic meridional overturning circulation (AMOC, Rahmstorf et al., 2015; Caesar et al., 2018) and by accelerating heat transport to higher latitudes (Gervais et al., 2018; Keil et al., 2020). A similar pattern of cooling in the North Atlantic (the so‐called North‐Atlantic warming hole trend—NAWH) is also observed in the last few decades (Caesar et al., 2018; Drijfhout et al., 2012; Josey et al., 2018; Piecuch et al., 2017; Robson et al., 2016) and was attributed to anthropogenic activity and specifically to GHGs emissions (Caesar et al., 2018; Chemke et al., 2020). The formation of the NAWH was shown to drive atmospheric circulation changes that affect the European, and more generally, the Northern Hemisphere's climate (Gervais et al., 2019; Haarsma et al., 2015).

The second largest source of anthropogenic climate forcing, after GHGs, is due to anthropogenic aerosols (Stocker et al., 2014). Aerosols, particles suspended in the atmosphere, are released due to anthropogenic activity and affect Earth's radiation budget by direct interactions with radiation and by their effects on cloud properties (Bellouin et al., 2020). Unlike GHGs, aerosols are inhomogeneously distributed in the atmosphere and are therefore generally assumed to drive corresponding regional changes to the atmospheric circulation (Allen et al., 2015; Chemke & Dagan, 2018). However, despite differences in forcing structure, previous studies demonstrated a similar spatial pattern of response of surface temperature to GHGs and aerosol forcing (with an opposite sign; Xie et al., 2013). Specifically, aerosol forcing was shown to strengthen the AMOC and hence to oppose the GHGs effect (Cai et al., 2006; Delworth & Dixon, 2006; Menary et al., 2020). The aerosol strengthening of the AMOC was explained by a combination of temperature and salinity effects on the sea water density in the North Atlantic (Delworth & Dixon, 2006). The preferential cooling of the Northern Hemisphere atmosphere by aerosol forcing leads to stronger heat flux from the ocean to the atmosphere, driving an increase in the surface water density. Concomitantly, the atmospheric cooling reduces the fresh water flux to the North Atlantic, which again increases the surface water density. The increased upper ocean density drives stronger oceanic convection and stronger AMOC (Delworth & Dixon, 2006).

In addition, aerosol forcing was proposed to dominate the North Atlantic surface temperature variability (Booth et al., 2012; Qin et al., 2020), while other studies highlighted internal variability as dominant source (Terray, 2012; Zhang et al., 2013). Here, analyzing data from CMIP6 models (Phase 6 of the Coupled Model Intercomparison Project) and CESM‐LE (the Community Earth System Model Large Ensemble), we show that the forcing due to anthropogenic aerosol opposes the formation of the NAWH, consistent with previous studies demonstrating aerosol‐induced AMOC strengthening (Cai et al., 2006; Delworth & Dixon, 2006; Menary et al., 2020). We quantify the delay of the appearance of the NAWH due to anthropogenic aerosol as about three decades.

## 2 Methods

### 2.1 CMIP6

We use data from the Coupled Model Intercomparison Project Phase 6, specifically from the DAMIP—Detection and Attribution Model Intercomparison Project (Gillett et al., 2016), the ScenarioMIP—scenario model intercomparison project (O'Neill et al., 2016) and the Historical runs. From DAMIP we present results from two different types of simulations—hist‐aer and hist‐GHG. Both simulate the historical period (1850–2020) with either only aerosol forcing (hist‐aer) or only GHGs forcing (hist‐GHG). In addition, we present results from the Historical simulations, simulating the full anthropogenic forcing for the historical period. From the ScenarioMIP we present two different future scenarios—ssp245 and ssp585, which represent medium and high anthropogenic radiative forcing projections at the end of the 21st century, respectively (O'Neill et al., 2016). The aerosol conditions used in CMIP6 and the induced radiative forcing and the intermodel spread during the historical and future periods are well documented by recent studies (Fiedler, Kinne, et al., 2019; Fiedler, Stevens, et al., 2019; Smith et al., 2020). For each model, for each scenario, one realization was used. The temperature anomalies are calculated compared to the preindustrial control simulations. The list of models included in each set of simulations can be found in Table S1 in the supporting information.

### 2.2 CESM‐LE

We also use data from the CESM‐LE (Kay et al., 2015), a set of 40 simulations (ensemble members) using CESM1 (Hurrell et al., 2013). Between 1920 and 2005 all simulations are subject to the historical forcing (Full‐forcing) and between 2006 and 2100 to the CMIP5 RCP8.5 scenario (which represent a relatively fast increase in GHGs; Riahi et al., 2011). In addition, 20 ensemble members were subject to the same forcing but with a fixed aerosol forcing corresponding to 1920 aerosol levels (fixAER; Deser et al., 2020). In the RCP8.5 scenario, during the 21st century the ratio between aerosol forcing and GHGs forcing decreases with time (Westervelt et al., 2015). The difference between the Full‐forcing and the fixAER represents the aerosol effect (keeping in mind that in this case the aerosol levels are set to 1920 levels and not to preindustrial levels as in DAMIP). The temperature fields in these ensembles are compared to an 1,800‐year‐long CESM preindustrial control run (constant 1850 forcing).

### 2.3 Observations

Two different observation‐based sea surface temperature (SST) data sets are included here: the Hadley Centre HadISST (Rayner et al., 2003) and the National Oceanic and Atmospheric Administration (NOAA) extended reconstructed SST V4 (Huang et al., 2015). The HadISST data are available on 1° resolution since 1870 and based on the Met Office Marine Data Bank. The NOAA SST data are on 2° resolution and available since 1854. For both data sets, the temperature anomalies are calculated as the difference from the mean between 1870 and 2020.

For all—CMIP6, CESM‐LE, and the observations, we examine the temporal evolution of the temperature anomalies difference between the region of the North Atlantic (43–60.5°N, 315–345°W) and a larger region around it (20–60.5°N, 300–345°W) to account for the local temperature contrast.

## 3 Results

**Figure 1:**
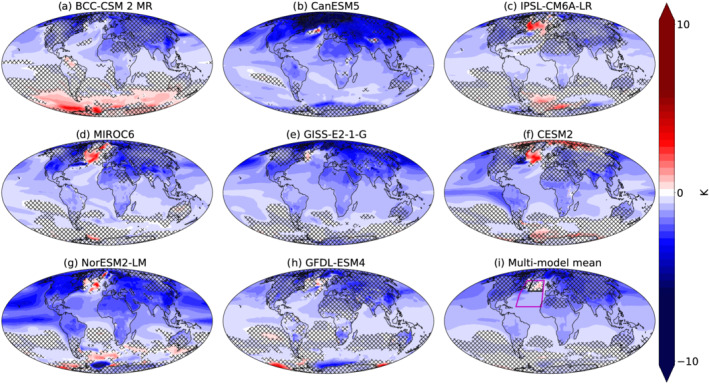
Temperature difference between the last 30 years of the CMIP6 hist‐aer simulations and the time mean preindustrial simulations for the different models (a–h) and the multimodel mean temperature change (i). The uncrossed areas are regions in which changes in temperature are statistically significant based on a *t* test (*p* value > 0.05).

We start by examining the surface temperature change in CMIP6 hist‐aer simulations (simulations of the historical period forced only by anthropogenic aerosol; that is, the GHG levels are as in preindustrial times—section 2) compared to the preindustrial control runs (the last 30 years of the historical period minus the average over the preindustrial period). Figure 1 demonstrates that, as expected, aerosol forcing generates a global cooling, which is stronger in the Northern Hemisphere, especially at mid and high latitudes. However, most (seven out of eight) of the models demonstrate a temperature increase in the North Atlantic, which is also shown in the multimodel mean. This pattern resembles the opposite of the temperature change pattern shown to emerge due to GHGs forcing (Chemke et al., 2020; Cheng et al., 2013; Drijfhout et al., 2012; Marshall et al., 2015; Rahmstorf et al., 2015; Woollings et al., 2012) and is referred to here as the aerosol‐driven North Atlantic cooling hole. We note that different models demonstrate slightly different location of the North Atlantic aerosol‐driven cooling hole in a similar manner to the location of the NAWH (Menary & Wood, 2018). The variation in the location of the cooling hole could be due to different model formulations (e.g., differences in subgrid parameterizations or external forcing) and, for the case of the location of the NAWH, was shown to be related to the different strength of the oceanic circulation and locations of deepwater formation in the different models (Menary & Wood, 2018). In this paper we present global maps to provide global perspective. This demonstrates that the warming in the North Atlantic is the only statistically significant warming pattern globally in the multimodel mean.

**Figure 2:**
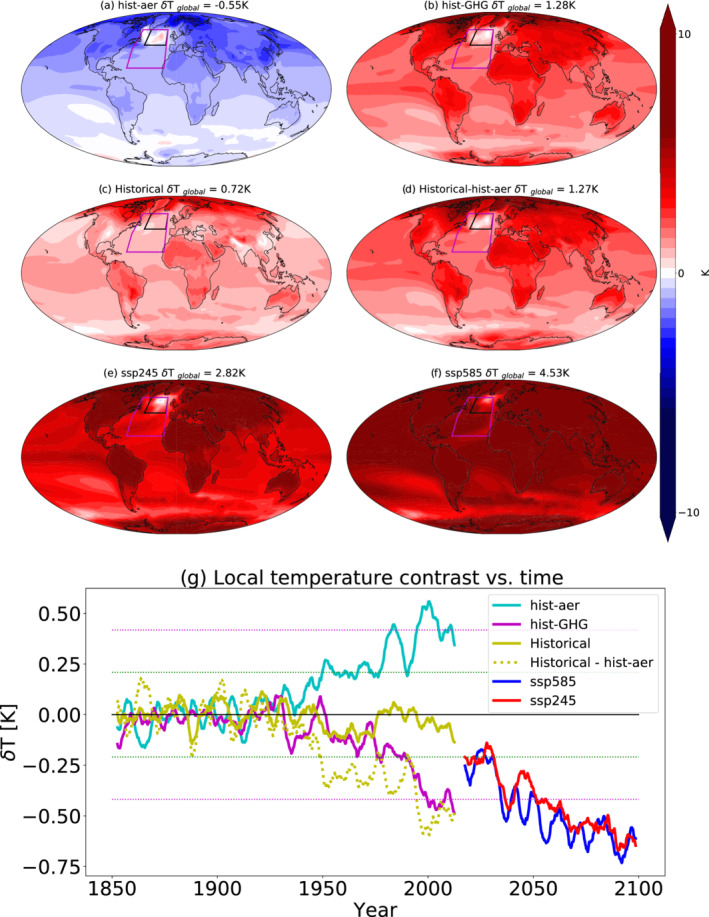
(a–f) The multimodel mean temperature difference between the last 30 years of the different CMIP6 scenarios ([a] hist‐aer, [b] hist‐GHG, [c] Historical, [d] Historical‐hist‐aer, [e] ssp245, and [f] ssp585) and the preindustrial simulations. (g) The 5‐year running mean multimodel mean difference in temperature anomalies (compared to the preindustrial) between the North Atlantic cooling hole region (marked in black in the maps) and its surroundings (marked in magenta) for the different scenarios. The green and magenta dotted lines represent, respectively, the 1 and 2 standard deviations of the multimodel mean temperature anomaly difference between these two regions in the preindustrial simulations. Also presented here is the difference between the Historical simulations and the hist‐aer simulations.

To quantify the aerosol effect on the NAWH compared to other anthropogenic forcing agents, we present in Figure 2 the time evolution of the 5‐year running mean temperature anomaly differences (section 2) between the North Atlantic region (43–60.5°N, 315–345°W—marked in black in Figure 2, referred to here as the North Atlantic cooling hole region) and a larger region surroundings it (20–60.5°N, 300–345°W—marked in magenta) under different scenarios (Historical, hist‐GHG, hist‐aer, and two future scenarios ssp245 and ssp585—section 2). We compare the temperature evolution in the North Atlantic cooling hole region to its surroundings to capture the local temperature contrast. A similar trend is seen when comparing the temperature difference between the North Atlantic cooling hole region and the global mean temperature anomalies or between the tropical Atlantic and the North Atlantic (Terray, 2012). In addition, Figure 2 presents maps of the multimodel mean temperature difference between the last 30 years of each scenario and the preindustrial simulations.

Figure 2 demonstrates that aerosol forcing generates a relative warming of the North Atlantic compared to its surroundings (a cooling hole), while GHGs generate the opposite trend (warming hole). A high similarity (with opposite sign) between the regional pattern of temperature change due to GHGs and aerosol forcing is noted (Xie et al., 2013). According to the metric used here, the NAWH does not emerge in the Historical runs and becomes significant (outside of the 2 standard deviations based on the temperature variability in the preindustrial control runs) only in the future scenarios (ssp245 and ssp585) toward the end of 2020s. Calculating the difference between the Historical runs and the hist‐aer (representing an historical scenario without aerosol forcing—assuming that the forcings are additive) demonstrates that without the aerosol forcing, the NAWH would have been apparent before the end of the twentieth century. We note that a similar trend (and global geographical temperature pattern) appears in the hist‐GHG simulations, suggesting that the forcings are broadly additive (as GHGs and aerosol are the two major anthropogenic forcing agents). These results suggest that the aerosol forcing delays the formation of the NAWH by about 30 years. We note that large uncertainties regarding the magnitude and spatial distribution of the aerosol forcing remain (Bellouin et al., ). A recent paper showed that the strength of the aerosol forcing in the different models and, in particular the representation of aerosol‐cloud interactions, can have a strong effect on the aerosol‐driven strengthening of the AMOC (Menary et al., 2020). Since the location of the warming hole is different in the different models (Figure 1), in Figure 2, we present the analysis of the local temperature contrast around its location in the multimodel mean.

**Figure 3:**
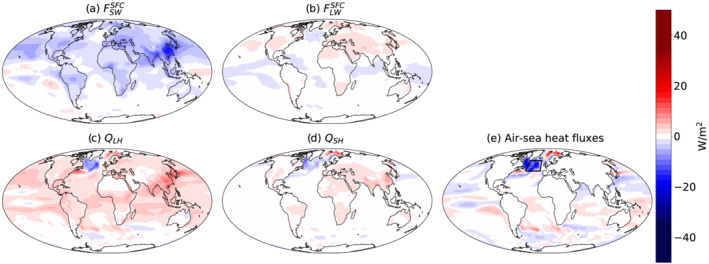
The CMIP6 multimodel mean difference in (a) surface radiative shortwave flux ( $F_{\mathrm{SW}}^{\mathrm{SFC}}$), (b) surface radiative longwave flux ( $F_{LW}^{\mathrm{SFC}}$), (c) latent heat (*Q*_*LH*_), and (d) sensible heat (*Q*_*SH*_), between the last 30 years of the hist‐aer simulations and the preindustrial simulations. Also presented (e) is the total air‐sea heat flux change, calculated as the sum of $F_{\mathrm{SW}}^{\mathrm{SFC}}$, $F_{\mathrm{LW}}^{\mathrm{SFC}}$, *Q*_*LH*_, and *Q*_*SH*_. Negative values are upward, that is, causing cooling of the surface.

A temperature increase in the North Atlantic due to aerosol forcing (Figures 1 and 2) could be caused either by air‐sea heat flux changes or by changes in the ocean heat flux (especially of the AMOC; Cai et al., 2006; Delworth & Dixon, 2006; Menary et al., 2020). Figure 3 presents the changes in all the different components of the air‐sea flux (sensible heat *Q*_*SH*_, latent heat *Q*_*LH*_, surface radiative longwave $F_{\mathrm{LW}}^{\mathrm{SFC}}$, and radiative shortwave flux $F_{\mathrm{SW}}^{\mathrm{SFC}}$ changes) and its sum. The main aerosol effect is to reduce $F_{\mathrm{SW}}^{\mathrm{SFC}}$ by direct interaction (scattering and absorbing) with the shortwave radiation in the atmosphere (Ramanathan et al., 2001) and by interaction with clouds (Albrecht, 1989; Twomey, 1974). The reduction in $F_{\mathrm{SW}}^{\mathrm{SFC}}$ drives a general decrease in the magnitudes of *Q*_*SH*_ and *Q*_*LH*_. Since *Q*_*SH*_ and *Q*_*LH*_ on average lead to surface cooling, the decrease in their magnitudes drives relative surface warming. Hence, Figures 3c and 3d demonstrate mostly positive values outside of the North Atlantic. The global maps presented in Figure 3 demonstrate the uniqueness of the local response over the North Atlantic compared to the general response (especially in the case of *Q*_*SH*_ and *Q*_*LH*_, which decrease in the North Atlantic but generally increase elsewhere). The decrease in *Q*_*SH*_ and *Q*_*LH*_ over the North Atlantic (stronger surface cooling) due to aerosol forcing is caused by the local increase in surface temperatures.

Figure 3 demonstrates that the air‐sea heat flux changes in the North Atlantic cooling hole region cannot explain the local increase in surface temperature and is even counteracting it (causing a local reduction in surface temperature over the North Atlantic). A similar compensating effect by air‐sea heat flux was shown before for the case of GHGs induces NAWH (Gervais et al., 2018; Menary & Wood, 2018). Hence, we conclude that the local increase in surface temperature over the North Atlantic is caused by changes in the ocean heat flux (in agreement with previous studies; Cai et al., 2006; Delworth & Dixon, 2006; Menary et al., 2020). This is also consistent with previous studies (Caesar et al., 2018; Chemke et al., 2020; Cheng et al., 2013; Drijfhout et al., 2012; Gervais et al., 2018; Marshall et al., 2015; Menary & Wood, 2018; Piecuch et al., 2017; Rahmstorf et al., 2015; Robson et al., 2016; Woollings et al., 2012) showing that the NAWH is driven by a reduction in meridional heat flux in the ocean. However, this analysis cannot distinguish between the contribution of an acceleration in the low‐latitude AMOC (Cai et al., 2006; Delworth & Dixon, 2006; Menary et al., 2020) and the declining contribution of heat transport to higher latitudes (Keil et al., 2020).

The CMIP6 hist‐aer data are based on only eight different models (that were available as of June 2020). Using model ensembles partially removes the spread due to the different model formulations (Figure 1); however, such a small ensemble may not account entirely for the natural variability of the system. The fact that not all the natural variability is eliminated is the likely cause of the oscillations in time seen in Figure 2 (Milinski et al., 2019). In order to examine the aerosol effect on the North Atlantic surface temperature and accounting for natural variability in the climate system we utilize the CESM‐LE (section 2) under the historical and future RCP8.5 scenarios with all forcings (Full‐forcing) and with fixed‐aerosol forcing (fixAER, cf. section 2). In a large ensemble of simulations forced by the same forcing, the ensemble members' mean eliminates natural variability and isolates the climate response to the external forcing (cf. section 2).

**Figure 4:**
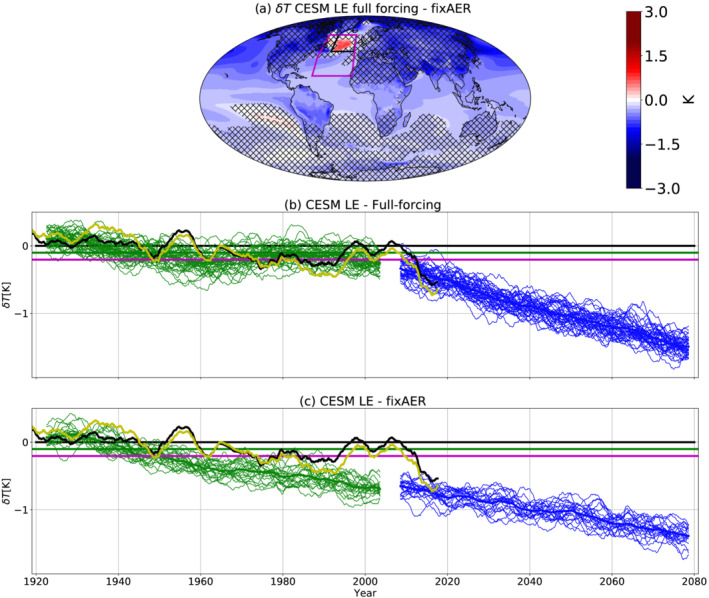
(a) The CESM‐LE mean temperature difference between the last 30 years of the full historical forcing (Full‐forcing, 1975–2005) and the fixAER forcing (representing the aerosol effect). (b and c) The 5‐year running mean difference in temperature anomalies (compared to the preindustrial) between the North Atlantic cooling hole region (marked in black in the map) and its surroundings (marked in magenta) in the Full‐forcing (b) and fixAER (c). Green lines represent the historical period, and blue lines the future RCP8.5 scenario. Thin lines represent the different ensemble members, while the thick lines represent the ensemble mean. Green and magenta horizontal lines represent, respectively, the one and two standard deviations of the temperature anomaly difference between these two regions in the preindustrial simulations. Uncrossed areas in (a) are regions in which changes in temperature are statistically insignificant based on a *t* test (*p* value > 0.05). Black and yellow curves in (b) and (c) present the same temperature difference from two observation‐based data sets: Hadley Centre HadISST (black) and the NOAA extended reconstructed SST V4 (yellow).

Examining the surface temperature difference between the full historical forcing and the fixAER (representing the aerosol effect; Figure 4) demonstrates, again, a pattern of global cooling (especially in the Northern Hemisphere) with a cooling hole (and even a warming) over the North Atlantic (Deser et al., 2020). We note that the aerosol driven North Atlantic cooling hole appears in the same location in CESM‐LE and the CMIP6 multimodel mean. The global maps presented in Figure 4 demonstrate, again, the uniqueness of the local warming in the North Atlantic compared to the global cooling. Here we follow again the 5‐year running mean time evolution of the temperature anomalies (compared to the preindustrial) in the North Atlantic cooling hole region (marked in black in Figure 4—similar region as in Figures 1, 2, 3) and its surroundings (marked in magenta) in the Full‐forcing runs and in the fixAER runs. It demonstrates that in both cases the ensemble mean temperature difference becomes negative with time (representing a warming hole). However, in the historical runs only in the last few years all ensemble members become negative, while in the fixAER simulations it is the case from the midtwentieth century. Adopting a similar criteria as in the CMIP6 case (temperature difference between the North Atlantic cooling hole region and its surroundings larger than 2 standard deviations of the CESM preindustrial runs—0.204 K) demonstrates that the NAWH emerges around 1990 (all ensemble members below 2 standard deviations of the preindustrial runs) for the fixAER runs and around 2020 for the future RCP8.5 Full‐forcing runs (and does not emerge during the historical period under the Full‐forcing ending at 2005). Similar to the CMIP6 case, aerosol forcing delays the formation of the NAWH in CESM‐LE by about 30 years.

We note that in both cases, Full‐forcing and fixAER, the temperature difference is slightly positive at the beginning of the time series (1920). This might be a result of the way these ensembles were constructed, running a single simulation between 1850 and 1920 and initiating the large ensemble based on the conditions in this specific simulation at 1920 (with each simulation differ by small random noise perturbations to their initial atmospheric temperature fields). Hence, it is to be expected that this single simulation does not have the same temperature fields as the preindustrial control run, with which the temperature anomalies are compared. In addition, we note that in the Full‐forcing case the ensemble mean temperature difference demonstrates a reduction between 1920 and 1960, followed by a recovery up to 1990. This trend is consistent with the strongest aerosol effect on the AMOC during the latter period (Menary et al., 2020).

According to these simulations, if there had been no aerosol forcing, the forced response of the NAWH could have been 0.3 K colder at the end of the historical period (2005; averaged over the region marked in black in Figure 4a), despite the faster global mean temperatures increase (the local temperature contrast, as presented in Figures 4b and 4c, would have been 0.45 K larger).

Figures 4b and 4c also present the time evolution of the same temperature‐anomaly difference from two different observation‐based data sets: the Hadley Centre HadISST (Rayner et al., 2003; black curve) and the NOAA extended reconstructed SST V4 (Huang et al., 2015; yellow curve). It demonstrates that the observations lie generally within the spread of the CESM‐LE. It also demonstrates that, in agreement with previous studies (Caesar et al., 2018; Chemke et al., 2020; Drijfhout et al., 2012; Josey et al., 2018; Piecuch et al., 2017; Robson et al., 2016), the NAWH emerged from the internal climate variability only recently. In addition, Figure 4c demonstrates that the fixAER runs are inconsistent with the observations and develop the NAWH too early and too strongly.

We note that the difference between the Full‐forcing and the fixed‐AER ensembles (Figures 4b and 4c) decreases during the 21st century due to the decrease in aerosol forcing with time. At the end of the 21st century, the RCP8.5 scenario is largely dominated by GHGs forcing (Westervelt et al., 2015). This demonstrates that the expected reduction in aerosol forcing during the 21st century will accelerate the formation of the NAWH.

## 4 Discussion

In this paper we document an increase in surface temperature in the North Atlantic under anthropogenic aerosol forcing based on state‐of‐the‐art climate models. This regional temperature increase occurs despite an aerosol‐driven global cooling, which is particularly pronounced in the Northern Hemisphere. This pattern resembles the opposite of the well documented pattern of the North Atlantic warming hole (NAWH) predicted under global warming (Chemke et al., 2020; Cheng et al., 2013; Drijfhout et al., 2012; Marshall et al., 2015; Rahmstorf et al., 2015; Woollings et al., 2012). We demonstrate that this cooling hole pattern in the North Atlantic cannot be explained by air‐sea heat flux changes and hence must involve changes in the ocean heat flux. This is with agreement with previous studies demonstrating aerosol induced strengthening of the AMOC (Cai et al., 2006; Delworth & Dixon, 2006; Menary et al., 2020). Aerosol forcing has been shown before to strengthen the AMOC by increasing the near surface water density due to preferential cooling of the Northern Hemisphere atmosphere, which leads to both stronger heat flux from the ocean to the air and weaker fresh water flux to the ocean (Delworth & Dixon, 2006).

We quantify the effect of aerosol on the NAWH compared to other anthropogenic forcing agents, in both CMIP6 models and the CESM‐LE by examining the local temperature contrast and show that it delays the formation of it by about 30 years. It should be noted that a recent study demonstrated that in CMIP6 models AMOC is more sensitive to aerosol forcing than in CMIP5 and in observations (Menary et al., 2020). However, the fact that CESM‐LE shows the same trend increases our confidence in the robustness of our results. In addition, we show here that the temporal evolution of the temperature contrast around the NAWH region, based on two different observation‐based SST data sets, falls within the ensemble range of CESM‐LE Full‐forcing. This increases our confidence in using CESM‐LE to study the NAWH and demonstrates that indeed, with aerosol forcing, the NAWH emerged from the internal climate variability only recently.

In future decades the aerosol forcing is expected to decline. Our results suggest that the predicted reduction in aerosol forcing will accelerate the formation of the NAWH, which could have important implications for the climate in the Northern Hemisphere (O'Reilly et al., 2017; Woollings et al., 2012; Zhang & Delworth, 2006).

## Supporting information

Supporting Information S1

## Data Availability

The data are available at the following websites: CESM‐LE (https://www.cesm.ucar.edu/projects/community‐projects/MMLEA/), CMIP6 (https://pcmdi.llnl.gov/CMIP6/), NOAA SST (https://www.esrl.noaa.gov/psd/), and HadISST (https://www.metoffice.gov.uk/hadobs/hadisst/).
