# Supplementary material for: Aerosol Forcing Masks and Delays the Formation of the North Atlantic Warming Hole by Three Decades
Source: Geophys Res Lett. 2020 Nov 18;47(22):e2020GL090778. doi: 10.1029/2020GL090778 (PMC7757191; doi:10.1029/2020GL090778)
Supplement: Supplementary file 1 — Supporting Information S1 [file GRL-47-e2020GL090778-s001.pdf]

Supporting information of:

Aerosol forcing masks and delays the formation of the North-Atlantic warming hole by three decades

Guy Dagan[1], Philip Stier[1], Duncan Watson-Parris[1]

[1] Atmospheric, Oceanic and Planetary Physics, Department of Physics, University of Oxford, UK

E-mail: guy.dagan@physics.ox.ac.uk

Content:

Table S1.

Table S1. List of the CMIP6 models analyzed in this study and the scenario they are used in.

Model	Modelling center	Hist-aer	Hist-GHG	Historical	Ssp585	Ssp425
BCC-CSM-2-MR	Beijing Climate Center, China Meteorological Administration	✓	✓	✓	✓	✓
CanESM5	Canadian Centre for Climate Modeling and Analysis	✓	✓	✓	✓	✓
CESM2	National Center for Atmospheric Research	✓	✓	✓		
CESM2-WACCM	National Center for Atmospheric Research				✓	✓
GFDL-CM4	NOAA Geophysical Fluid Dynamics Laboratory			✓		
GFDL-ESM4	NOAA Geophysical Fluid Dynamics Laboratory	✓	✓		✓	✓
GISS-E2-1-G	NASA Goddard Institute for Space Studies	✓	✓	✓		
IPSL-CM6A-LR	Institut Pierre-Simon Laplace	✓	✓	✓	✓	✓
MIROC6	Atmosphere and Ocean Research Institute (The University of Tokyo), National Institute for Environmental Studies, and Japan Agency for Marine-Earth Science and Technology	✓	✓	✓	✓	✓
NorESM2-LM	Norwegian Climate Centre	✓	✓		✓	✓
NorESM2-MM	Norwegian Climate Centre			✓		
